# Alarming development of dual snus and cigarette usage among young Finnish males

**DOI:** 10.1186/s12889-019-7519-1

**Published:** 2019-09-11

**Authors:** Maria Danielsson, Anelma Lammi, Simo Siitonen, Jukka Ollgren, Liisa Pylkkänen, Tuula Vasankari

**Affiliations:** 1 The Finnish Defence Forces, Fabianinkatu 2, 00130 Helsinki, Finland; 2 Doctoral School in Health Sciences, University of Helsinki, P.O. BOX 3, 00014 Helsinki, Finland; 3 Finnish Lung Health Association (FILHA), Filha Ry, Sibeliuksen katu 11 A 1, 00250 Helsinki, Finland; 4 National Institute for Health and Welfare, P.O. BOX 30, 00271 Helsinki, Finland; 5 Finnish Medicine Agency Fimea, Helsinki, Finland; 6 Division of Medicine, Department of Oncology, Turku University Hospital, and University of Turku, P.O. Box 52, 20521 Turku, Finland; 7 Division of Medicine, Department of Pulmonary Diseases and Clinical Allergology, Turku University Hospital, and University of Turku, P.O. Box 52, 20521 Turku, Finland

**Keywords:** Tobacco, Cigarettes, Smokeless tobacco, Snus, Dual use, Transition, Education

## Abstract

**Background:**

The consumption of tobacco products has evolved to include more complex combinations of different products. We investigated the tobacco habits of a representative population of young Finnish male conscripts in order to evaluate the prevalence of dual use of cigarettes and snus as well as the transition from one tobacco product to another. In addition, we evaluated the correlation between the level of education and the use of cigarettes and snus.

**Methods:**

A questionnaire-based survey was carried out in three out of 17 garrisons among conscripts during their first week of service in 2014. A total of 1971 male conscripts were selected by simple random sampling of the 9013 males in the selected garrisons. Of them 1916 participated and filled in the questionnaire. The response rate was 97.2%. The questionnaire consisted of 25 questions including age, gender, basic education, use of tobacco products as well as questions assessing nicotine dependency.

**Results:**

The amount of dual users of cigarettes and snus was 21%. There was a higher probability of dual use of cigarettes and snus among smokers compared to snus users (*p* < 0.001). One third (35%) of former smokers reported daily snus use and over 40% of the former snus users smoked daily. One third (34%) of the participants reported snus usage and 14% of the study subjects used snus daily. 40% of the study population were smokers and over 25% smoked daily. Of the participants with basic educational background 57% smoked daily (*p* < 0.001), however, no association between snus and level of education was found (*p* = 0.69).

**Conclusions:**

This study provides better understanding of the complex tobacco habits of young adult males. The simultaneous usage of multiple tobacco products as well as the high tendency to transition from one tobacco product to another should be taken into consideration when planning cessation interventions in health care settings and tobacco control policies at societal levels.

**Electronic supplementary material:**

The online version of this article (10.1186/s12889-019-7519-1) contains supplementary material, which is available to authorized users.

## Key points

- Exclusive snus use as well as dual use of cigarette and snus were significantly higher than expected.
- Smoking correlated with low educational level, but this could not be shown among snus users.
- The notably high prevalence of snus usage seems to reflect the emerging change of trend among tobacco consumers.

## Background

A significant decline in smoking, especially among well-educated individuals can be seen worldwide in developed countries [1–3]. However, there are signs that the use of non-cigarette tobacco products has increased despite tobacco control programs, restriction on marketing of tobacco products and the ban on  trade of smokeless tobacco in Australia, Israel and the European Union, excluding Sweden [3, 4]. The consumption of tobacco products has evolved to include more complex tobacco habits and the dual use of different tobacco products, mainly cigarettes and smokeless tobacco, has become common [5–11].

The transition from the Swedish type low- nitrosamine smokeless tobacco (snus) to cigarettes and vice versa has mainly been studied in Sweden. According to Galanti et al. (2008), who analyzed several studies from Sweden, Finland and North America, current snus use was mainly associated with non-smokers or former smokers. Smoking induction or the gateway theory only seemed to affected a minority [12]. However, Furberg et al. (2006) discovered that the transition from cigarettes to snus use among males is often incomplete resulting in dual usage [13]. Some research suggests that smokeless tobacco might actually initiate smoking [14–16].

Snus is most commonly used in Scandinavia and the United States, prominently in Sweden and Norway, where smokeless tobacco trade is legal [17, 18]. A study Hamari et al. (2013) carried out on military recruits in Northern Finland indicated that almost half of daily snus users also smoked [8]. In Sweden, Norway and in the United States dual usage is relatively uncommon, but the pattern is more frequent among young adults and adolescents [7, 19, 20]. Recent studies also show increased awareness, openness and readiness for consuming non-cigarette tobacco products [11, 20–25].

Snus is perceived as a less harmful product than cigarettes and even as a means of harm reduction [26, 27], but the absorption through the mucous membrane is effective and results in a high dose of nicotine intake. In addition, snus contains a high amount of nicotine, around 20 carcinogens and over 2500 chemicals [17, 28].

The correlation between disadvantaged socioeconomic and socio-educational status and smoking is well acknowledged [29–32]. This seems to apply to the transition from sporadic use to daily use as well [33]. However, knowledge about the correlations between snus use and socio-educational status is limited. Some studies suggest that use of snus is more often related to favorable social and health profiles than daily smoking [19, 31].

As discussed above, recent developments point towards a transition from exclusive smoking to usage of other tobacco products and complex tobacco habits. We therefore evaluated the prevalence of dual use of cigarettes and snus as well as the transition from one tobacco product to another by reported quitters, among a representative population of young Finnish males entering military service. In addition, we examined the overall prevalence of cigarette and snus usage and the correlation between the educational background of the participants and their smoking habits.

## Methods

### Study population

All Finnish males must attend a call-up for military service the year they turn 18 years of age and attend service before the age of 30. Some conscripts may be excluded from military service due to medical or unsuitability factors or they may choose to do non-military service [34–36]. Approximately 77% of men from the age group attend military service, while about 2% of females voluntarily choose to do so [37, 38].

### Sampling

Three out of 17 Finnish garrisons, The Guard Jaeger Regiment, the Karelian Brigade and the Kainuu Brigade were chosen as they train recruits coming from different parts of Finland. The troops were selected by simple random sampling.

**Fig. 1:**
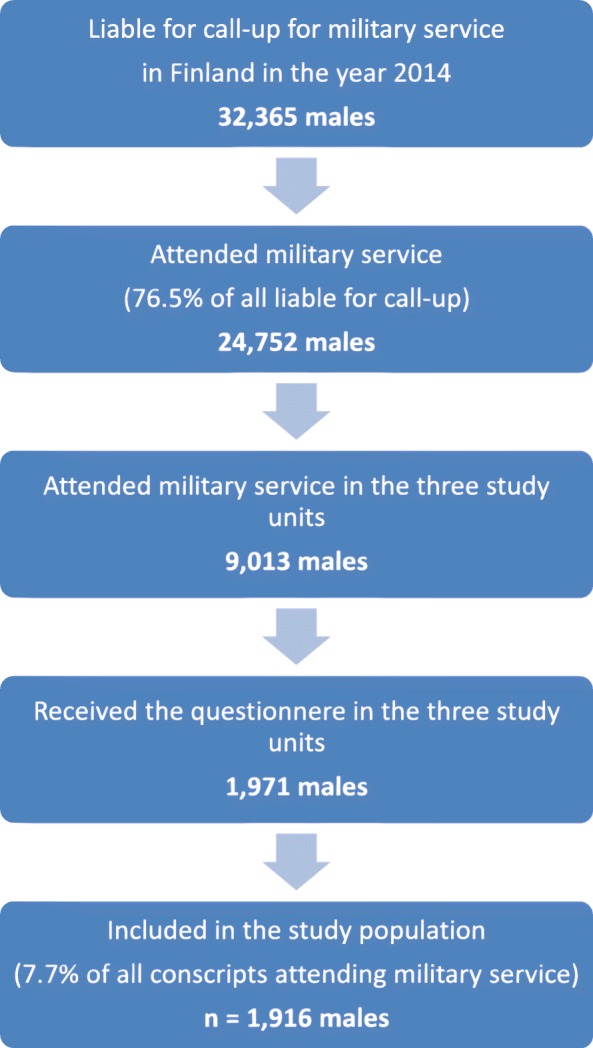
Flowchart showing the data selection of the study population in the year 2014

This study covered both cohorts entering military service in January and June 2014. Altogether 9013 males of those 24,752 Finnish males attending military service during the study year started their service in the selected three garrisons, from which we by simple random sampling chose a representative sample of 1971 male conscripts of which 1916 participated and filled in the questionnaire. The response rate was high, 97.2%. Figure 1.

The material reflects the tobacco habits of young adult Finnish males. Females were excluded from this analysis because only 2.7% (*n* = 53) of the respondents were women and could not be considered to represent the use of tobacco products of Finnish young women in general.

### Data collection and measurement

The questionnaire-based survey was carried out concurrently with the general health inspection during the first week of service. The questionnaire developed for this study consisted of 25 questions including age, gender, basic education, use of tobacco products including tobacco and snus as well as questions assessing nicotine dependency. The use of electric cigarettes was included in the questionnaire but was excluded from the analysis due to very low usage (1.4%). Additional file 1

Tobacco users were grouped according to their tobacco habits. The three main categories were smokers, snus users and non-users. Self-rolled tobacco was combined with manufactured tobacco due to the very low number of self-rolled users. These groups were subdivided into daily users, occasional users, quitters and dual users of cigarettes and snus. The ‘never’-group was defined by the statement “I have never smoked or used snus on a daily basis”. Questions concerning smoking and snus use were formulated as recommended by WHO and validated in several earlier studies [39].

The dual use of cigarettes and snus was determined by the simultaneous daily and/or occasional use of both products. The habit of consumption was not specified. Transition from one product to another was calculated by comparing current smokers/snus users to current quitters.

The educational level was subdivided into the following three categories: basic education (consisting of 9 years of compulsory education), vocational school and upper secondary school.

### Statistical analysis

The data was analyzed by using the IBM SPSS Statistics software package, version 23. The Chi-Square Test of independence was used to assess if there is a relationship between two categorical variables. Trends in a larger-than-2 × 2 table with ordinal level categorical variables were tested with the Mantel-Haenszel test of trend (the linear-by-linear association test). The equality between the table row marginal proportions and the corresponding table column proportions (marginal homogeneity) were tested by the marginal homogeneity test. [40–42]

### Ethical approval

The study was approved by the Ethical Committee of Helsinki and Uusimaa Hospital District, Finland. All participants gave their written informed consent. The subjects were informed about the use of the collected data for research purposes, and consented to participate on a voluntary basis in compliance with the principles of the WMA Declaration of Helsinki.

## Results

The mean age of the study population of 1916 male conscripts was 19.4 years (±SD 1.1 years) with a range of 18–28 years. A majority, 92% of the study subjects, were 18–20 years. Half (51%) of the respondents had attended upper secondary school, 38% had a vocational education background and 10% had only basic education prior to conscription. Table 1.

**Table 1:** Daily use of cigarettes, snus and dual use of both products according to educational status (*n* = 1911) among the study population of Finnish male conscripts in 2014

	Daily smokers^1^	Daily snus users^2^	Daily dual use
Basic education	113 (56.8%)	27 (13.6%)	10 (5%)
Vocational school	282 (38.8%)	108 (15.0%)	22 (3%)
Upper secondary school	96 (9.9%)	132 (13.6%)	12 (1.2%)
All	491 (25.9%)	267 (14.2%)	44 (2.3%)

Linear-by-linear association test for the trend: ^1^*p* < 0.001, ^2^ *p* = 0.690

### Use of any tobacco products

Almost 40% of these conscripts were smokers, either daily or occasionally, and of them 66% smoked every day. Respectively, every third young male (34%) used snus and 42% of them used it daily. Table 2.

**Table 2:** Smoking, use of snus and dual use of cigarettes and snus among Finnish male conscripts in 2014 (*N* = 1916)

Tobacco usage	Daily snus user	Occasional snus user	Former snus user	No snus use	Total
Daily smoker	45 (2.4%)	201 (10.6%)	52 (2.8%)	191 (10.1%)	489 (25.8%)
Occasional smoker	74 (3.9%)	82 (4.3%)	16 (0.9%)	80 (4.2%)	252 (13.3%)
Former smoker	56 (3.0%)	20 (1.1%)	31 (1.6%)	51 (2.7%)	158 (8.4%)
Non-smoker	92 (4.9%)	64 (3.4%)	22 (1.2%)	816 (43.1%)	994 (52.5%)
Total	267 (14.1%)	367 (19.4%)	121 (6.4%)	1138 (60.1%)	1893 (100%)

Linear by linear association test for the trend: *p* < 0.001

Our findings showed a strong linear association between the educational background of the participants and their smoking habits (*p* < 0.001). Only 10% of the subjects with an upper secondary educational background smoked daily, while 57% of those with a basic education smoked every day. However, we could not find a statistically significant correlation between educational level and exclusive snus use (*p* = 0.690). Table 1.

### Dual use of tobacco products

In our study, up to 21% of all conscripts reported dual tobacco consumption and over 2% reported daily use. 9% of the daily smokers used snus every day and 41% occasionally. Respectively, 17% of daily snus users smoked daily and 28% occasionally. Table 2.

The dual use of cigarettes and snus had a linear association and are hence not independent factors (linear-by-linear test, *p* < 0.001). There was a higher probability of dual use of cigarettes and snus among smokers compared to snus users. Table 2.

### Transition from one tobacco product to another

Our results showed that, 8% of the male conscripts had quit smoking, and of these 35% used snus daily, 13% occasionally, and 20% had quitted the use of snus.

In total 6% of all participants had stopped using snus. Of these, 43% smoked daily, 13% occasionally, and 26% had quit smoking. Table 3.

**Table 3:** Transition from one tobacco product to another after quitting either smoking or snus use among young Finnish male conscripts

	Former smokers (*N* = 158)	Former snus users (*N* = 121)
	Do you use snus now?	Do you currently smoke?
Daily	56 (35.4%)	52 (43.0%)
Occasionally	20 (12.7%)	16 (13.2%)
Used earlier, but have quitted	31 (19.6%)	31 (25.6%)
No transition to another tobacco product after quitting	51 (32.3%)	22 (18.2%)

Cf. Table 2

### Additional analysis

We studied the robustness of the results represented in the chapters above in respect to age distribution by excluding the older age groups from the analysis. Age groups 19 years and 19–21 years were analyzed separately. The results were coherent with the whole study material.

### Summary of key results

Our results showed that one third of the participants reported snus usage, of which 42% used snus daily (i.e 14% of the study subjects). Smoking was also more common than expected. One fourth of the study population smoked daily, while as many as 40% reported sporadic or regular cigarette consumption. The dual use of cigarettes and snus was surprisingly high, with 21% of all conscripts reporting dual usage. Interestingly, about one third of former smokers reported daily snus usage and over 40% of reported quitters in the snus-using group smoked daily - showing a transition to another tobacco product instead of quitting.

## Discussion

We analyzed the prevalence and dual use of cigarettes and snus, as well as transition from one tobacco product to another, among young male adults at the beginning of their military service. We found that dual consumption of cigarettes and snus was common, especially among daily smokers as many as half reported simultaneous snus usage. Interestingly, about one third of former smokers reported daily snus usage and over 40% of reported quitters in the snus-using group smoked daily - showing a transition to another tobacco product instead of quitting.

Limited data is available regarding dual use of cigarettes and snus or transition behavior, particularly in this target group. However, Scandinavian and Northern American studies imply that snus usage is growing among young adults. Dual usage is still uncommon and is mostly associated with current smoking. The prevalence of exclusive snus usage is high in Sweden and Norway where the trade of smokeless tobacco is legal [7, 18, 20, 43, 44].

Our study results are in line with the study conducted on recruits in Northern Finland by Hamari et al.(2013) [8]. Furthermore, the School Health Promotion Study indicated that the dual use of cigarette and snus is currently gaining popularity among Finnish adolescents in their late teens [11]. Some dual users might be at a transitional stage, switching from one product to another, but it is noteworthy that the probability of continuing dual usage is high [6, 7, 45].

We observed that the transition from one tobacco product to another among reported quitters was very common. About one third of former smokers reported daily snus usage. Over 40% of reported quitters in the snus-using group smoked daily instead. For a more accurate understanding of the phenomenon, it would have been valuable to determine the time elapsed in the transition, as well as the time-span after quitting smoking. Unfortunately, we did not collect this data. Based on our findings, the prevalence of both snus usage and smoking was significantly higher than in the Finnish national health surveys [11, 21, 24, 46]. Daily smoking was twice as common in our study and snus usage was three times more common than in the adolescent health and lifestyle survey [21, 24]. On the other hand, the corresponding use of snus among Finnish army recruits in Northern Finland has been shown earlier. This trend is worrying considering that the trade of smokeless tobacco is banned in the European Union (excluding Sweden) and might reflect a wider international trend [3, 8, 44]. Finland’s geographical vicinity to Sweden makes illegal trade compelling.

Large population-based health examination surveys provide a good overall understanding of the tobacco habits of adolescents and the adult population, although the response rates have decreased and rarely exceed 70%. The exceptionally high response rate of over 97% gained in our study might explain the marked difference in the prevalence of smoking between studies.

Smoking was common among the study subjects with a basic educational background. The difference was clear compared to the study subjects with an upper secondary education, of whom only 10% smoked daily. Snus usage was equally common for all educational levels. Our results confirm previous findings associating lower educational status and smoking in Northern European countries [29]. However, we could not confirm any association between the level of education and snus use. This association has not been clear in earlier studies either, although some studies have shown a less prominent association among snus users [19, 47].

The strength of our study was the ability to carry it out in a military setting and to reach a comprehensive portion of young adult males, since all adult Finnish males are liable for military service. The survey was conducted upon arrival during the general health inspection, which motivated the conscripts to answer the questionnaire. The response rate was significantly higher than in most national surveys. Answering the survey was restricted to the first week of the military service to ensure responses that reflected the tobacco habits before the military culture had influenced them.

A common concern is selection bias. Young smoking men with a low education are typically under-represented [24, 46, 48–50]. A minority of young men are exempted from military service due to suitability or health issues, such as mental health problems, causing a possibility of bias in our results. We do not know the prevalence of tobacco use among these individuals, but we may assume that the prevalence of tobacco usage is likely to be even higher among the non-selected population. As for women, military service is voluntary and only a few women are recruited for service. We therefore excluded women from the results due to the low number of participants.

## Conclusions

Our findings showed that exclusive snus use as well as dual usage of cigarettes and snus was significantly higher than expected from previous studies. A statistically significant correlation between educational level and exclusive snus usage could not be demonstrated.

This study provides a better understanding of the complex tobacco habits of young adult males, who are an important target population for health interventions. Young adults are at a transitional and vulnerable age when the usages of tobacco products, as well as other health habits, are yet to be established. The simultaneous usage of multiple tobacco products as well as the high tendency of transition from one tobacco product to another, should be taken into consideration when planning cessation interventions in health care settings and tobacco control policies at societal levels.

## Additional file

Additional file 1: The questionnaire used to conduct the survey regarding the tobacco habits among conscripts. (DOCX 24 kb)

## Data Availability

The datasets generated and/or analysed during the current study are not publicly available due to unpublished material that will be used in future publications but are available from the corresponding author on reasonable request.

## Abbreviations

WHO: World Health Organization
WMA: World Medical Association
