# Supplementary material for: Alarming development of dual snus and cigarette usage among young Finnish males
Source: BMC Public Health. 2019 Sep 11;19:1249. doi: 10.1186/s12889-019-7519-1 (PMC6737716; doi:10.1186/s12889-019-7519-1)
Supplement: Supplementary file 1 — The questionnaire used to conduct the survey regarding the tobacco habits among conscripts. (DOCX 24 kb) [file 12889_2019_7519_MOESM1_ESM.docx]

**A survey on tobacco habits among conscripts**

1. How old are you?

_______ years

1. What is your gender?
   1. male
   2. female
2. Where do you live permanently?

____________________________

1. How long is your military service?
   1. 165 days
   2. 255 days
   3. 347 days
   4. I don’t know
2. What is your level of education?
   1. high school
   2. vocational education
   3. upper-secondary
3. How many cigarettes or cigars have you smoked during your entire life? How many times have you smoked tobacco in a pipe during your entire life?
   1. none / never
   2. just one / just once
   3. 2-50 / times
   4. over 50 / times
4. Do you currently smoke (cigarettes, cigars or tobacco in a pipe)?
   1. yes, every day
   2. sometimes
   3. I have quit
   4. I have never smoked on a regular basis
5. Have you ever tried snus? How many times up until now?
   1. never
   2. just once
   3. 2-50
   4. over 50
6. Do you use snus now?
   1. yes, every day
   2. sometimes
   3. I’m trying to quit or have quit
   4. I have never used snuff on a regular basis
7. How many of the following products do you use every day? Fill in each blank. Put in 0 if you do not smoke at all.
   1. manufactured cigarettes _____ a day
   2. cigarettes you roll yourself _____ a day
   3. snus ______ times a day
   4. electronic cigarette ______ times a day
8. If you smoke cigarettes or use snus, how long have you been using these products?
   1. smoked _____ years
   2. used snus _____ years
9. Do regular personnel smoke in the presence of conscripts?
   1. yes, every day
   2. yes, sometimes
   3. no
   4. I don’t know
10. Do regular personnel use snus when conscripts are present?
    1. yes, every day
    2. yes, sometimes
    3. no
    4. I don’t know
11. If you currently smoke, would you like to quit?
    1. Yes
    2. No
12. If you currently use snus, would you like to quit?
    1. Yes
    2. No
13. If you smoke and/or use snus, what type of support would help you the MOST to quit smoking/using snus?
    1. peer support online (for e.g. Facebook groups, discussion forums, nurse online)
    2. a one-on-one meeting with a health care professional
    3. support group meetings
    4. information and training on the effects of smoking / using snuff
    5. the support of friends and family
    6. some other type of support
    7. I do not need support to stop smoking / using snus
14. If you smoke or/and use snus, has one of the persons listed below recommended in the past twelve months that you quit smoking / using snus?

| a doctor | yes | no |
| --- | --- | --- |
| a nurse or health care  professional at work | yes | no |
| your dentist | yes | no |
| a pharmacist | yes | no |
| a family member | yes | no |
| someone else | yes | no |

1. How many times have you stopped smoking in the past six months?
   1. I have never smoked regularly
   2. I have not smoked in the past six months
   3. not once
   4. yes, once
   5. yes, twice or more
2. How many times have you stopped using snus in the past six months?
   1. I have never used snuff regularly
   2. I have not used snuff in the past six months
   3. not once
   4. yes, once
   5. yes, twice or more
3. If you want to quit smoking, do you intend to quit in the near future?
   1. I don’t smoke
   2. I don’t want to quit smoking
   3. I will not try quitting smoking in the near future
   4. yes, in the coming 30 days
   5. yes, sometime in the next 2-6 months
   6. yes, sometime in the next 7-12 months
   7. yes, in over a year’s time
4. If you want to stop using snus, do you intend to stop in the near future?
   1. I don’t use snus
   2. I don’t want to stop using snus
   3. I will not attempt to stop using snus in the near future.
   4. yes, in the coming 30 days
   5. yes, sometime in the next 2-6 months
   6. yes, sometime in the next 7-12 months
   7. yes, in over a year’s time
5. If you do smoke, how quickly within minutes will you have your first cigarette after waking up?
   1. in less than 6 minutes
   2. 6-30 minutes
   3. 31-60 minutes
   4. over 60 minutes
6. If you do use snus, how quickly within minutes will you use snus after waking up?
   1. in less than 6 minutes
   2. 6-30 minutes
   3. 31-60 minutes
   4. over 60 minutes
7. What is your opinion on smoking and using snus?

|  | Not at all | Hardly | Neither good or bad | Clearly | Very damaging |
| --- | --- | --- | --- | --- | --- |
| How damaging to your health is smoking? | 1 | 2 | 3 | 4 | 5 |
| How damaging to your health is snus? | 1 | 2 | 3 | 4 | 5 |
| How addictive are cigarettes? | 1 | 2 | 3 | 4 | 5 |
| How addictive is snus? | 1 | 2 | 3 | 4 | 5 |

1. What is your experience of the following situations?

|  | Not at all | Seldom | Sometimes | Often | Always |
| --- | --- | --- | --- | --- | --- |
| May conscripts smoke when they are performing their military service? | 1 | 2 | 3 | 4 | 5 |
| May regular personnel smoke during working hours? | 1 | 2 | 3 | 4 | 5 |
| Do people smoke in military service / during working hours? | 1 | 2 | 3 | 4 | 5 |
| May conscripts use snus when they are performing their military service? | 1 | 2 | 3 | 4 | 5 |
| May regular personnel use snus during working hours? | 1 | 2 | 3 | 4 | 5 |
| Is snus used during military service / working hours? | 1 | 2 | 3 | 4 | 5 |
| Have there been situations where smoking has been an advantage while in the army (e.g. during breaks)? | 1 | 2 | 3 | 4 | 5 |
| Have there been situations where using snus has been an advantage while in the army (e.g. during breaks)? | 1 | 2 | 3 | 4 | 5 |

**THANK YOU FOR COMPLETING THE SURVEY!**
